# Galectin-3 promotes brain injury by modulating the phenotype of microglia via binding TLR-4 after intracerebral hemorrhage

**DOI:** 10.18632/aging.205014

**Published:** 2023-09-11

**Authors:** Tianyu Liang, Zheng Zhu, Fangxiao Gong, Xiaobo Yang, Xiaoju Lei, Ling Lu

**Affiliations:** 1 Emergency and Critical Care Center, Intensive Care Unit, Zhejiang Provincial People’s Hospital (Affiliated People’s Hospital), Hangzhou Medical College, Hangzhou, Zhejiang, China; 2 Center for General Practice Medicine, Department of Nursing, Zhejiang Provincial People’s Hospital (Affiliated People’s Hospital), Hangzhou Medical College, Hangzhou, Zhejiang, China; 3 Center for Rehabilitation Medicine, Department of Anesthesiology, Zhejiang Provincial People’s Hospital (Affiliated People’s Hospital), Hangzhou Medical College, Hangzhou, Zhejiang, China

**Keywords:** intracerebral hemorrhage, galectin-3, neuroinflammation, microglia, TLR-4

## Abstract

Background: Intracerebral hemorrhage (ICH) is a stroke subtype with high mortality and disability rate, and neuroinflammation is involved in secondary brain injury. Galectin-3 (Gal-3) is one of the scaffold proteins of Galectins. Studies have indicated that Gal-3 plays an important role in the physiological and pathological state of the nervous system. Here we focus on the role of Gal-3 in ICH, especially in neuroinflammation.

Methods: Injection of autologous blood into the right basal ganglia was used to simulate ICH injury, and the level of Gal-3 in brain was regulated by related means. The changes of Gal-3 were detected by western blot and immunofluorescence, the level of neuroinflammation by immunofluorescence staining and ELISA. Apoptosis and neuron loss were detected by TUNEL staining FJB staining and Nissl staining, and neurological deficits were judged by neurobehavioral tests.

Results: The protein level of Gal-3 increased at 24 h after ICH. Downregulation of Gal-3 level can reduce the infiltration of M1-type microglia and peripheral inflammatory cells, thus alleviating post-ICH neuroinflammation, and reducing cell apoptosis and neuron loss in brain tissue. ICH-induced neurological damage was rescued. Meanwhile, the promotion in the expression level of Gal-3 increased neuroinflammatory activation and nerve cell death, aggravating ICH-induced brain injury.

Conclusions: This study proves that Gal-3 is involved in neuroinflammation and nerve damage after ICH. Gal-3 expression should not be encouraged early on to prevent neuroinflammation. which provides a new possibility for clinical treatment for ICH patients.

## INTRODUCTION

As the second most prevalent stroke subtype, intracerebral hemorrhage (ICH) is a critical condition with high mortality and morbidity rates [1, 2]. The one-month case fatality rate of cerebral hemorrhage is 40%, and the one-year rate is 54%. Only 12%-39% of survivors achieve favorable outcomes. Although minimally invasive neurosurgery has made progress in acute interventions, early hematoma clearance, and resolving mass effects in the past decade, ICH mortality and disability rates remain elevated [3, 4]. Survival and recovery from cerebral hemorrhage depend on hematoma location, mass effect, increased intracranial pressure from potential hematomas, subsequent cerebral edema due to neurotoxicity or inflammation surrounding the hematoma, and complications from long-term neurological dysfunction [5–7]. Identifying effective ICH treatments and, more importantly, strategies to mitigate ICH-related harm is essential for determining the extent to which ICH patients can return to societal life.

Galectins are carbohydrate-binding proteins with conserved carbohydrate-recognition domains (CRDs) and galactoside binding capabilities. They participate in numerous biological processes, including migration, adhesion, growth, apoptosis, immune response, and cell survival [8, 9]. Galectin-3 (Gal-3), a member of the galectin family, features a single C-terminal CRD and an N-terminal domain containing a collagen-like internal repeat domain, enabling it to form pentamers [10]. Numerous studies have demonstrated Gal-3’s role as a potential cytokine regulating inflammation in various diseases [11–13]. Gal-3’s significance in modulating immune response and inflammation, as well as its role in the transition from acute to chronic inflammation, has been well-established [14]. Recent research has confirmed that Gal-3 promotes the sustained transformation of macrophages into pro-inflammatory phenotypes, and activated microglia can secrete Gal-3, resulting in elevated Gal-3 expression levels in many brain disorders [15, 16]. Moreover, it has been shown that Gal-3 drives neuroinflammation via binding TLR-4 [17]. We reasonably speculate whether Gal-3 will bind with TLR-4 to exert an inflammatory regulatory effect, and have designed relevant experiments to confirm. Therefore, investigating Gal-3’s role and related pathway following ICH is crucial.

Currently, there are no studies on Gal-3 in ICH, and research on Gal-3 and neuroinflammation, macrophage/microglia system is limited; thus, further examination of these relationships is necessary. As an essential regulator, Gal-3 is anticipated to play a significant role in neurological disorders. By studying Gal-3 changes after ICH and verifying its function, it is hoped that a novel target for future clinical treatments can be provided.

## MATERIALS AND METHODS

### Animals

Adult male Sprague-Dawley (SD) rats (weight 280-320g; Animal Center of Chinese Academy of Sciences, Shanghai, China) were accommodated in an environment with a temperature of 18-26° C, humidity of 40-70%, noise below 85 decibels, ammonia concentration below 20PPm, ventilation of 8-12 times/hour under a standard 12-hour light/dark cycle. Food and water were supplied without restriction. In order to “optimize” animal experimental strategies, provide experimental animals with humane care and to reflect animal welfare and the “3R” principle ((reduction, replacement, and refinement) in animal experiments, the “humane endpoints” was established, including: 1. Quickly shed pounds; 2. Appetite loss; 3. Exceptionally weak;4. Serious illnesses; 5. Visibly depressed mental state; 6. Organ malfunction; 7. Contagious illnesses, chronic wounds, severe hypothermia, etc. Anyway, we strived to minimize the number of animals used and the pain of the experimental process. The procedures of execution were carried out in accordance with the euthanasia guidelines, and cervical dislocation was performed after deep anesthesia.

### Establishment of the experimental ICH model *in vivo*

**Figure 1:**
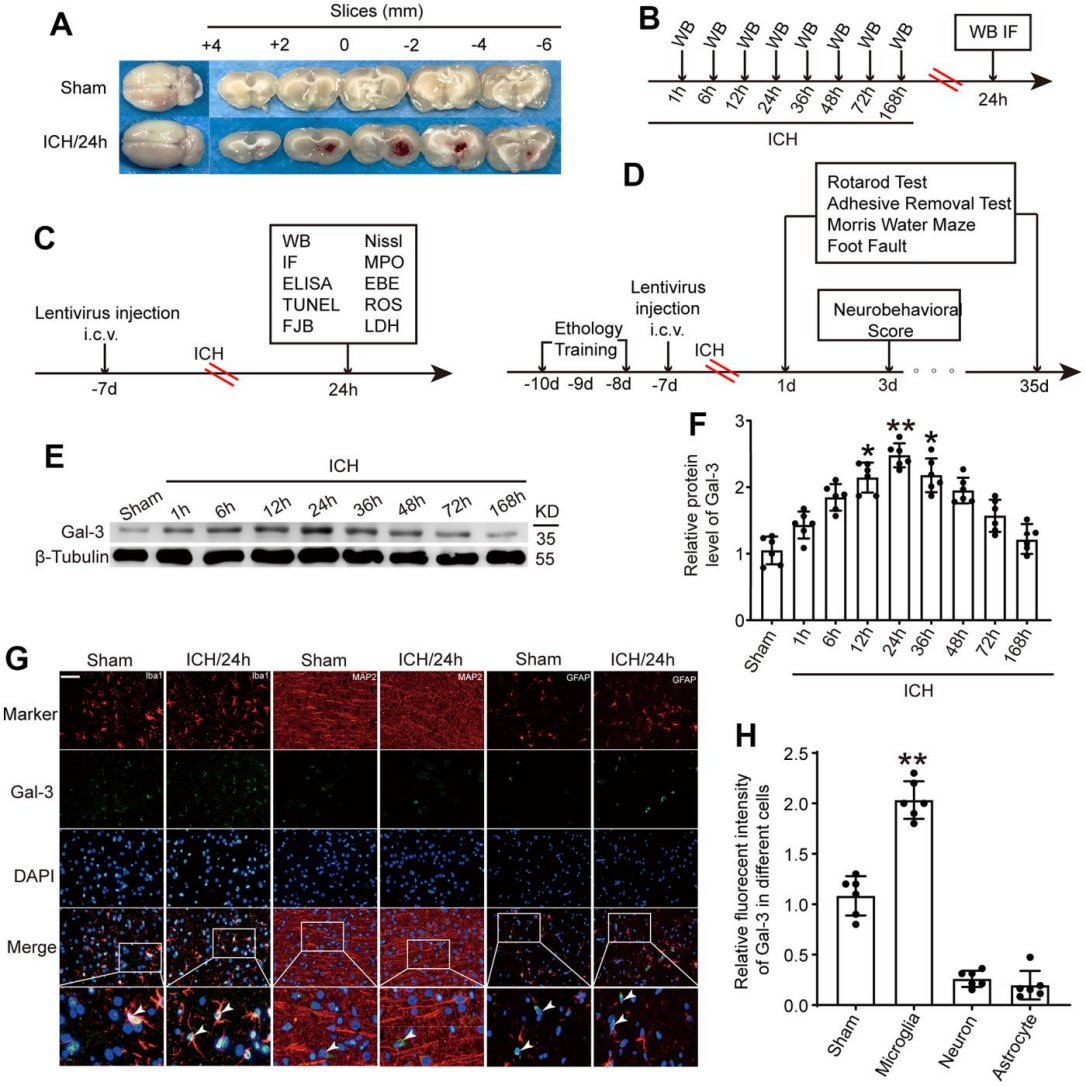
**The protein level of Gal-3 in microglia increased significantly at 24 h after ICH.** (**A**) Brain tissue sections of rats in sham group and intracerebral hemorrhage group. (**B**) Experiment 1: the time course changes of Gal-3 after ICH. (**C**, **D**) Experiment 2: the role of Gal-3 in ICH - induced brain injury. (**E**, **F**) Western blot analysis and quantification of Gal-3 at 1h, 6h, 12h, 24h, 36h, 48h, 72h, and 168h after ICH. (**G**, **H**) Double immunofluorescence analysis of Gal-3(green) and different brain cells (red) in brain sections. microglial marker (Iba-1)/Neuron marker (MAP2)/ Astrocyte marker (GFAP). Nuclei were labeled with DAPI (blue). Arrow indicated Gal-3 positive cells. Scale bar =50 μm. The black dots represent individual data in each group. **p < 0.01 and *p < 0.05 vs. sham group, n = 6.

Utilizing the injection of autologous blood into the basal ganglia, the ICH model was established in SD rats (Figure 1A). Experimental rats underwent anesthesia through an intraperitoneal administration of 4% chloral hydrate (400mg/kg) and were subsequently fixed in a prone posture on the stereotaxic apparatus. Following skin disinfection, a midline incision was performed to expose the skull. Employing a grinder, a cranial aperture was created above the right basal ganglia (3.5 mm rightward and 0.2 mm posterior to the bregma). Subsequently, 70 μl of autologous blood, procured from the cardiac region, was gradually introduced into the right basal ganglia by means of a microinjector (Hamilton Co, Reno, NV, USA) at a rate of 14 μl/min, achieving a puncture depth of 5.5 mm. After a 5-minute interval, the microinjector was carefully withdrawn. Upon needle extraction, bone wax was applied to occlude the burr hole, and the incision site was sutured. Experimental rats were then allowed to recuperate on a 37° C heated blanket. In the sham group, an equivalent volume of physiological saline solution was introduced into the right basal ganglia region.

### Experimental design

In this investigation, all SD rats were indiscriminately allocated to experimental cohorts, which remained undisclosed to the investigators. The study chiefly comprised the subsequent two segments.

In the first experiment, alterations in Gal-3 post- ICH were examined (Figure 1B). The rats were arbitrarily apportioned into seven clusters: sham assembly, and 1h, 6h, 12h, 24h, 36h, 48h, 72h and 168h after ICH, with 6 rats in each cohort. Western blot and immunofluorescence analyses were employed to ascertain the temporal fluctuations of Gal-3 after ICH.

In the second experiment, the focus was on exploring Gal-3’s role following ICH (Figure 1C, 1D). Overexpressed lentiviruses and small interfering RNAs were utilized to specifically modulate Gal-3 levels. A total of 161 rats were assigned to the following six groups: Sham, ICH, ICH+ shRNA-control, ICH+shRNA-Gal-3, ICH+LV-control, and ICH+LV-Gal-3 (n = 24 per group). After confirming the intervention’s efficacy using Western blot, various inflammatory indices were examined to elucidate Gal-3’s role in neuroinflammation, followed by further exploration of the ensuing cellular apoptosis and neural functional impairment.

### Transduction of lentivirus

The lentivirus employed in this investigation was supplied by Genechem company in Shanghai, China. During surgery, a small burr hole was drilled in the skull with the coordinates of 1.5 mm posteriorly and 1.0 mm laterally relative to the bregma. A 10 μl microliter syringe was mounted on the frame and the needle was inserted 3.5 mm below the bregma into the right lateral ventricle for the further injection of the lentivirus vectors. The titers of Gal-3 overexpression and Gal-3-RNAi lentiviruses are 1E+9 TU/ml and 8E+8 TU/ml, respectively. Five days prior to ICH, transfection was executed via lateral ventricular injection, and the transfection efficacy was verified by Western blot.

### Western blot

The Western blot analysis was performed as described previously [18]. Post-anesthesia, rats were administered phosphate-buffered saline, and RIPA lysis buffer was utilized to homogenize the brain tissue surrounding the hematoma. The homogenate samples were incubated on ice for 30 minutes and subsequently centrifuged at 12,000g at 4° C for 30 minutes. Equal protein quantities were separated using SDS-PAGE gel electrophoresis and transferred to a nitrocellulose membrane. The membrane was blocked with 5% BSA at room temperature for 1 hour, followed by overnight incubation with the primary antibody at 4° C. Densities were quantified using Image J software (National Institutes of Health, USA).

The primary antibody was purchased from ABCAM (ab76466, Dilution: 1:2000).

### Immunofluorescence staining

Immunofluorescence staining was performed according to previous study [19]. Cerebral tissue was preserved using 4% paraformaldehyde and subsequently fashioned into paraffin-embedded sections. The samples were heated at 70° C for 2 hours and underwent dewaxing through xylene, 95% ethanol, and 80% ethanol immersion. Antigen retrieval was achieved utilizing sodium citrate. A 5% BSA solution was employed for one-hour room temperature blocking, followed by overnight 4° C incubation with the primary antibody targeting the protein of interest. Upon washing the sections with PBST, the corresponding secondary antibody was introduced and incubated for 1.5 hours at 37° C in dark conditions. After mounting with DAPI, samples were visualized using a fluorescence microscope. The primary antibody was purchased from ABCAM (ab76466, Dilution: 1:100).

### Co-immunoprecipitation

The CO-IP procedure was carried out as previously illustrated. For the western blotting, the brain sample was prepared as instructed [20]. For western blotting and IP, the brain samples were first lysed in cell lysis buffer. Second, each mixture was incubated for 3 hours at 4° C with either rabbit IgG (as a negative control) or Gal-3 antibody. The protein-antibody-bead mixture was then incubated for 8 hours at 4° C with rotary agitation after each group of the protein mixture had been added Protein A/G Plus-Agarose. Third, the mixtures were denatured with 20 l 2 SDS loading buffer after being washed five times with cell lysis buffer. The relative protein levels of TLR-4 were then discovered using western blotting.

### Enzyme-linked immunosorbent assay (ELISA)

Enzyme-linked immunosorbent assay (ELISA) was utilized to quantify inflammatory cytokine concentrations in cerebrospinal fluid (CSF) and serum [21], including tumor necrosis factor-alpha (TNF-α), interleukin-1 beta (IL-1β), and interleukin-6 (IL-6). Following complete rat anesthesia, blood was collected from the heart and CSF procured via foramen magnum puncture. Commercial ELISA kits facilitated detection, adhering strictly to product guidelines.

### TdT-mediated dUTP-biotin nick end labeling (TUNEL) staining

TUNEL was used to evaluate cell apoptosis after ICH [22]. In simple terms, after the paraffin brain sections were deparaffinized and dehydrated, they were incubated with TUNEL reaction solution at 37° C for 1 h. After washing the slides with PBS, the slides were mounted with DAPI. TUNEL positive cells were observed under a fluorescence microscope, and apoptotic cells appeared green.

### FJC staining

The initial procedure mirrored that of immunofluorescent research. The pieces were heated and dewaxed, then incubated for 5 minutes in 80% alcohol with 20% sodium hydroxide, 2 minutes in 70% alcohol, 2 minutes in distilled water, 10 minutes in 0.06% K permanganate, and 20 minutes in 0.0004% FJC-working solution. They were then dried in an incubator (50–60° C) for 15–30 minutes. The pieces were dried and then incubated in xylene for two minutes. After that, neutral gum was used to secure them. Finally, a fluorescent microscope was used to view the sections.

### Nissl staining

Nissl staining was conducted as previously reported [23]. Deparaffinized and dehydrated brain sections were incubated with 0.5% toluidine blue at 37° C for 30 minutes, then sealed with neutral resin and observed using an optical microscope. Neuronal degeneration in the hippocampal CA1 region and cortex was evaluated by identifying shrunken, intensely stained neurons, indicative of degeneration and loss.

### Blood brain barrier (BBB) impairment

BBB impairment was measured by evans blue extravasation [24]. Following the prescribed therapies, the right femoral vein was promptly injected with evans blue in saline (2%, 3 ml/kg). After 60 minutes, rodents were given 1,000 mg/kg of urethane for deep anesthesia before being transcardially infused with ice-cold PBS until the fluid emanating from the right atrium was colorless. After rapidly embedding the same area in OCT embedding medium as for the brain water content assay, 7-m-thick cryosections of the tissue were cut. The slices were then captured by a fluorescence microscope and covers lipped with a water-based mounting medium. The remaining brain cells were then taken out and homogenized in icy PBS. Trichloroacetic acid was then added to precipitate protein, and the samples were cooled and centrifuged. A spectrophotometer was used to quantify the supernatant’s absorbance of evans blue at 610 nm.

### ROS analysis

Cerebral ROS concentrations served as an oxidative stress indicator, assessed via an ROS assay kit (Beyotime, China) [25]. Brain tissue samples underwent homogenization and centrifugation at 12,000 g for 10 minutes at 4° C, with supernatants collected. ROS levels were determined using the oxidant-sensitive probe 2,7-dichlorofluorescein diacetate (DCF-DA), with fluorescence intensity measured using a fluorometric microplate reader (Molecular Devices, USA) set at an excitation of 485 nm and emission of 530 nm. ROS concentrations in various groups were expressed as fluorescence intensity/mass of total protein (mg), and all samples’ ROS levels were normalized to those in the Sham group.

### LDH assay

The levels of LDH in CSF were determined using a specific LDH assay kit (Jiancheng Biotech, China). Results were presented corresponding to the relevant standard curves [26].

### Brain water content measurement

Employing the wet-dry method, as detailed in a previous study [27], cerebral edema indices were evaluated. Sodium pentobarbital was intraperitoneally administered 72 hours post-ICH induction, and the intact brain was immediately extracted. Brains were divided into two hemispheres along the midline and further dissected into five subregions: ipsilateral basal ganglia (Ipsi-BG), ipsilateral cortex (Ipsi-CX), contralateral basal ganglia (Cont-BG), contralateral cortex (Cont-CX), and cerebellum (CB). The respective sections were promptly weighed to record their wet weights, followed by dehydration at 100° C for 72 hours, after which their dry weights were measured. The water content proportion was calculated using the formula: [(wet weight - dry weight) / wet weight] × 100%.

### Neurological behavior scores

The modified Garcia scores were employed to assess neurological deficits (Table 1). This scoring system comprises seven tests: spontaneous activity, body proprioception, tentacle response, lateral bending, forelimb walking, limb symmetry, and climbing. Each item is worth 3 points, totaling 21 points, with lower scores indicating more severe neurological injury. Scores were measured at 3 days post-ICH model.

**Table 1:** Neurobehavioral evaluation: neuroscore scoring criteria for the sub-tests.

**Category**	**Behavior**	**Score**
Spontaneous Activity (SA)	Animal was akinesitic	0
	Animal moves slowly or minimally	1
	Animal approached 1-2 walls	2
	Animal approached at least 3 walls of the cage or raised on hindlimbs to explore the top of the cage	3
Vibrissae Proprioception (VP)	-	0
	Animal had a unilateral response	1
	Animal had either a weak bilateral response or weak left response and brisk right response	2
	Animal had a brisk bilateral response	3
Axial Senation (AS)	-	0
	Animal had no response on left side	1
	Animal had either a weak bilateral response or weak left response and brisk right response	2
	Animal had a brisk bilateral response	3
Limb Symmetry (LS)	Hemiparesis	0
	Left forelimb or left hindlimb flexed	1
	Asymmetric extension	2
	All limbs were extended symmetrically	3
Lateral Turning (LT)	Animal had no turning at all on one side	0
	Animal had unequal turning	1
	Animal turned bilaterally less than 45° on both sides	2
	Animal turned bilaterally at least 45^°^ on both sides	3
Forelimb Walking (FW)	Animal had a paretic forelimb	0
	Animal walked in circles	1
	Animal walked asymmetrically or to one side	2
	Animal briskly walked symmetrically on forepaws	3
Climbing (CL)	-	0
	Animal failed to climb or circled instead of climbing	1
	Animal climbed to the top and had a weak grip or animal climbed but had a strong grip	2
	Animal climbed to the top and had a strong grip	3

### Rotarod

Rotarod tests evaluated rats’ post-ICH motor function [28]. The rotating rod’s speed increased from 5 rpm to 40 rpm at an acceleration of 0.5 rpm/min. The latency time was recorded as the duration each rat remained on the rotating rod. Rats from each group were trained for 3 days before modeling, with pre-modeling data recorded as the “Pre value.” Tests were conducted at 1, 3, 5, 7, 10, 14, 21, 28, and 35 days after ICH.

### Foot fault

To appraise sensorimotor coordination, foot fault experiments were conducted utilizing a metallic grid measuring 45cm x 45cm with 2.5cm x 2.5cm grid cells and a height of 50cm. Rats were positioned at the grid’s center, and the left forelimb steps and foot fault steps were documented for 60 seconds. The proportion of foot fault steps to total steps served as the basis for statistical evaluation. Assessments were carried out on days 1, 3, 5, 7, 10, 14, 21, 28, and 35 after ICH.

### Adhesive removal

To quantify sensory functions, an adhesive removal assay was performed as previously outlined. A 9mm circular sticker was affixed to the inner portion of the rat’s left upper limb palm, and the time required for the rat to remove the sticker was recorded, with a maximum duration of 60 seconds. Assessments were conducted on days 1, 3, 5, 7, 10, 14, 21, 28, and 35 after ICH.

### Morris water maze

Finally, to appraise the long-term spatial learning and memory capabilities of rats, the Morris water maze experiment was executed from days 29 to 34 after ICH. A circular pool with a diameter of 180cm was employed, and a transparent circular platform, 20cm in diameter, was placed 1.5cm below the water surface in the fourth quadrant. The rat was situated in the second quadrant, and its trajectory in seeking the concealed platform was documented. The rat’s swimming speed, distance, and time were calculated for statistical analysis, with a total test duration of 60 seconds. On day 35 after ICH, we removed the platform and recorded the frequency of rats passing through the area where the platform was located and the time obtained by staying in the target quadrant.

### Statistical analyses

All data were reported as the mean SD and analyzed using GraphPad Prism8 software. Statistical differences between two groups were analyzed using the unpaired t-test and differences across multiple groups were analyzed using a one-way or two-way ANOVA. In all analysis, *p* <0.05 was considered statistically significant.

### Data availability

The data that support the findings of this study are available from the corresponding author upon reasonable request.

### Consent for publication

All authors have read the manuscript and approved for publication.

## RESULTS

### General observation

Body weight, body temperature, respiration, blood pressure, blood glucose, and other vital indicators were similar amongst the ICH groups, according to the monitoring data. In the Sham group, no rats perished. In the ICH groups in this study. The ICH death rate was 12.5% (24/192). The specific number of dead rats in each group and the time of death are presented in Table 2. The results were consistent with the ICH model death of SD rats approved by the national Ethical Committee in Animal Experimentation (CEEA, Comité d’Ethique en Experimentation Animale), from the French Ministry for Education and Research [29]. Due to ICH modeling, 24 rats perished. There was no significant difference in rat mortality rates between the ICH groups in Experiment 1 and Experiment 2, indicating that the ICH model construction by the researchers was stable. In Experiment 2, injection of interfering reagents had no significant impact on the mortality rate of ICH rats. We speculate that the causes of animal death may include the following: 1. The speed of blood injection was not balanced, and occasionally the speed was too fast, which led to the increase of intracranial pressure, and finally led to the death of the rats. 2. Errors in stereotaxic orientation caused blood to enter the cerebral ventricle and other areas, accelerating the death of rats. 3. Intracranial infection due to inadequate disinfection. Figure 1A displays typical coronal slices from rats in the Sham surgery group and the ICH group.

**Table 2:** Mortality in ICH-group.

**Group**	**Total**	**Death** **number**	**Survival number**	**Mortality** **rate**	**Death Time**
ICH-1h	7	1	6	14.3%	0.5h after surgery
ICH-6h	7	1	6	14.3%	2h after surgery
ICH-12h	6	0	6	0	Not applicable
ICH-24h	7	1	6	14.3%	1h after surgery
ICH-36h	7	1	6	14.3%	3h after surgery
ICH-48h	7	1	6	14.3%	3h after surgery
ICH-72h	7	1	6	14.3%	6h after surgery
ICH-168h	7	1	18	14.3%	2h after surgery
ICH	27	3	24	11.1%	2h after surgery; 5h after surgery; 7h after surgery
ICH+ shRNA-control	27	3	24	11.1%	1h after surgery; 8h after surgery; 13h after surgery
ICH+shRNA-Gal-3	28	4	24	14.2%	2h after surgery; 3h after surgery; 6h after surgery; 11h after surgery
ICH+LV-control	27	3	24	11.1%	0.5h after surgery; 3h after surgery; 4h after surgery
ICH+LV-Gal-3	28	4	24	14.2%	2h after surgery; 3.5h after surgery; 6h after surgery; 7h after surgery

### Gal-3 was upregulated in microglia after ICH

To ascertain endogenous Gal-3 fluctuations after ICH, brain tissue surrounding the hematoma was subjected to western blot analysis at 1h, 6h, 12h, 24h, 36h, 48h, 72h and 168h after ICH. Compared to the sham operation group, Gal-3 protein levels rose at 12h after ICH, peaked at 24h, and then gradually declined (Figure 1E, 1F). Given the extensive expression of Gal-3 in microglia, immunofluorescence co-staining of Gal-3 and microglial marker (Iba-1)/Neuron marker (MAP2)/ Astrocyte marker (GFAP) was conducted to determine Gal-3 protein levels. Results indicated Gal-3 is mainly expressed in microglia and the protein level of Gal-3 was dramatically increased in microglia surrounding the hematoma at 24h after ICH, corroborating the previous western blot findings (Figure 1G, 1H).

### Exogenous intervention effectively interfered with the expression of Gal-3 and Gal-3 binds to TLR-4 after ICH

**Figure 2:**
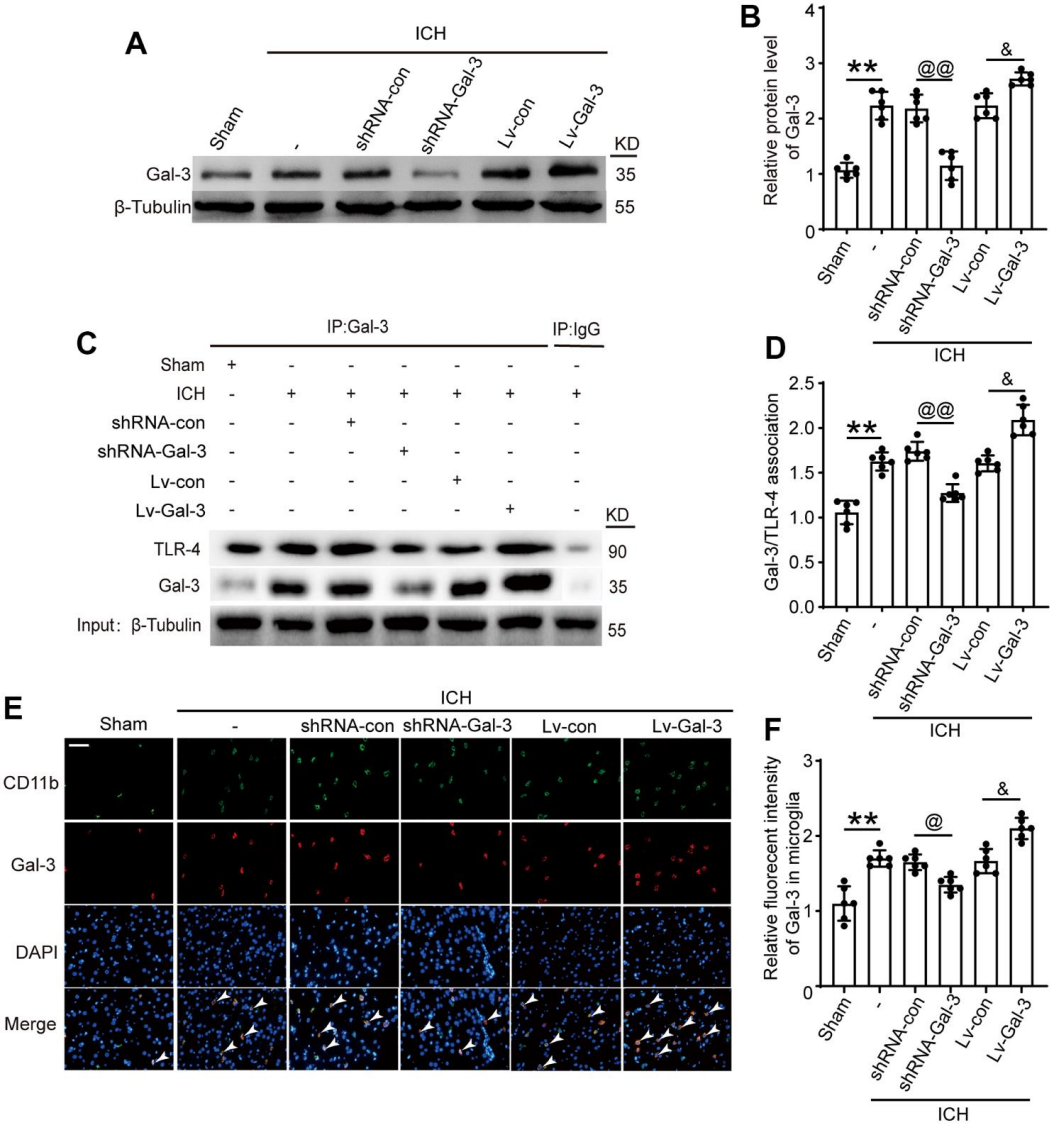
**Intervention efficiency of overexpression and small interfering RNA lentivirus on Gal-3.** (**A**, **B**) Western blot analysis and quantification of Gal-3 in intervention groups. (**C**, **D**) Gal-3/TLR-4 interactions in brain tissues after ICH and quantitative analysis was performed. (**E**, **F**) Double immunofluorescence analysis of Gal-3 (green) and microglia (red) in brain and the relative fluorescent intensity of Gal-3 in microglia, arrow indicated Gal-3 positive cells. Nuclei were labeled with DAPI (blue). Scale bar =50 μm. The black dots represent individual data in each group. **p < 0.01 and *p < 0.05 vs. Sham group, ^@@^*p* < 0.01 and ^@^*p* < 0.05 vs. ICH+shRNA-con group, ^&^*p* < 0.05 vs. ICH+Lv-con group, n = 6.

To further investigate Gal-3’s role in microglia and neuroinflammation following ICH, we employed lentiviral transfection to selectively downregulate and upregulate Gal-3. As depicted in Figure 2A, 2B, 2E, 2F, compared to the shRNA control group, the shRNA-Gal-3 group exhibited a significant decrease in Gal-3 protein levels; conversely, the LV-Gal-3 overexpression group displayed a notable increase in Gal-3 protein levels. Co-Ip experiments showed that when the expression level of Gal-3 increased after ICH, the protein level of TLR-4 bound to Gal-3 also increased sharply, while when the expression level of Gal-3 decreased, the level of TLR-4 bound to Gal-3 decreased accordingly (Figure 2C, 2D). These results indicate that TLR-4 and Gal-3 bind to play a related role after ICH.

### Gal-3 regulated the activation of inflammatory response in ICH-induced injury

**Figure 3:**
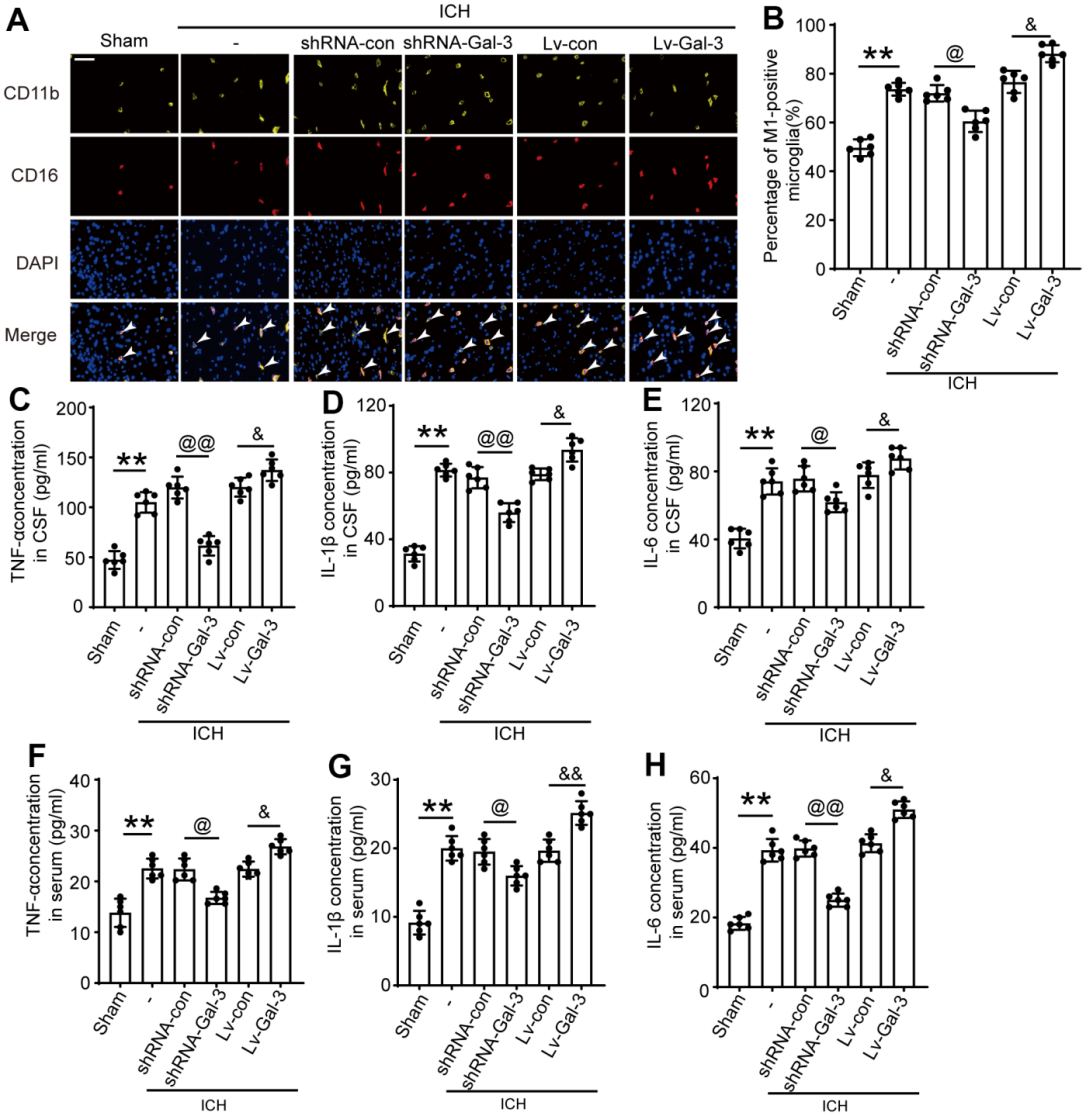
**Gal-3 aggravated the inflammatory response after ICH.** (**A**, **B**) Double immunofluorescence analysis of microglia(yellow) and M1-microglia (green) in brain and the percentage of M1-positive microglia was analysed, arrow indicated M1-microglia positive cells. (**C**) TNF-α in CSF. (**D**) IL-1β in CSF. (**E**) IL-6 in CSF. (**F**) TNF-α in serum. (**G**) IL-1β in serum. (**H**) IL-6 in serum. Nuclei were labeled with DAPI (blue). Scale bar =50 μm. The black dots represent individual data in each group. **p < 0.01 and *p < 0.05 vs. Sham group, ^@@^*p* < 0.01 and ^@^*p* < 0.05 vs. ICH+shRNA-con group, ^&&^*p* < 0.01 and ^&^*p* < 0.05 vs. ICH+Lv-con group, n = 6.

Subsequently, we examined the impact of Gal-3 on microglia after ICH. Numerous studies have demonstrated that microglial activation in the central nervous system primarily differentiates into M1 and M2 phenotypes, with M1 phenotype expressing pro-inflammatory cytokines that mediate neuroinflammation and M2 phenotype attenuating inflammation and repairing damage. CD16 is frequently employed as an M1 phenotype microglial marker; thus, we conducted CD11b (microglial marker) and CD16 immunostaining to investigate Gal-3’s influence on microglial polarization. Experimental outcomes revealed increased M1 phenotype microglia after ICH, while Gal-3 overexpression amplified M1 phenotype polarization and inflammation. In contrast, the shRNA-Gal-3 group exhibited reduced M1 phenotypes after ICH, suggesting that Gal-3 is involved in microglial M1 phenotype transformation following ICH and mediates neuroinflammation (Figure 3A, 3B).

Simultaneously, we conducted ELISA assays on rat cerebrospinal fluid (CSF) and plasma to measure inflammatory cytokine levels, including IL-1β, TNF-α, and IL-6, to further evaluate Gal-3’s impact on the inflammatory response following ICH. CSF ELISA results revealed significant increases in IL-1β, TNF-α, and IL-6 levels after ICH, with even more pronounced elevations upon Gal-3 overexpression, indicating heightened neuroinflammation. However, Gal-3 downregulation led to reduced IL-1β, TNF-α, and IL-6 levels, inhibiting inflammation activation (Figure 3C–3H). These findings further corroborate Gal-3’s crucial role in neuroinflammation following ICH. Moreover, plasma ELISA outcomes resembled those of CSF, suggesting a close association between rat ICH-induced neuroinflammation activation and systemic inflammatory response. While modulating neuroinflammation, it also impacts systemic inflammation levels. In the LV-Gal-3 group, plasma IL-1β and TNF- Both α and IL-6 were increased, and in the shRNA-Gal-3 group that down-regulated Gal-3, the levels of the three inflammatory cytokines were all decreased.

### Gal-3 was involved in regulating apoptosis and neuron loss after ICH

**Figure 4:**
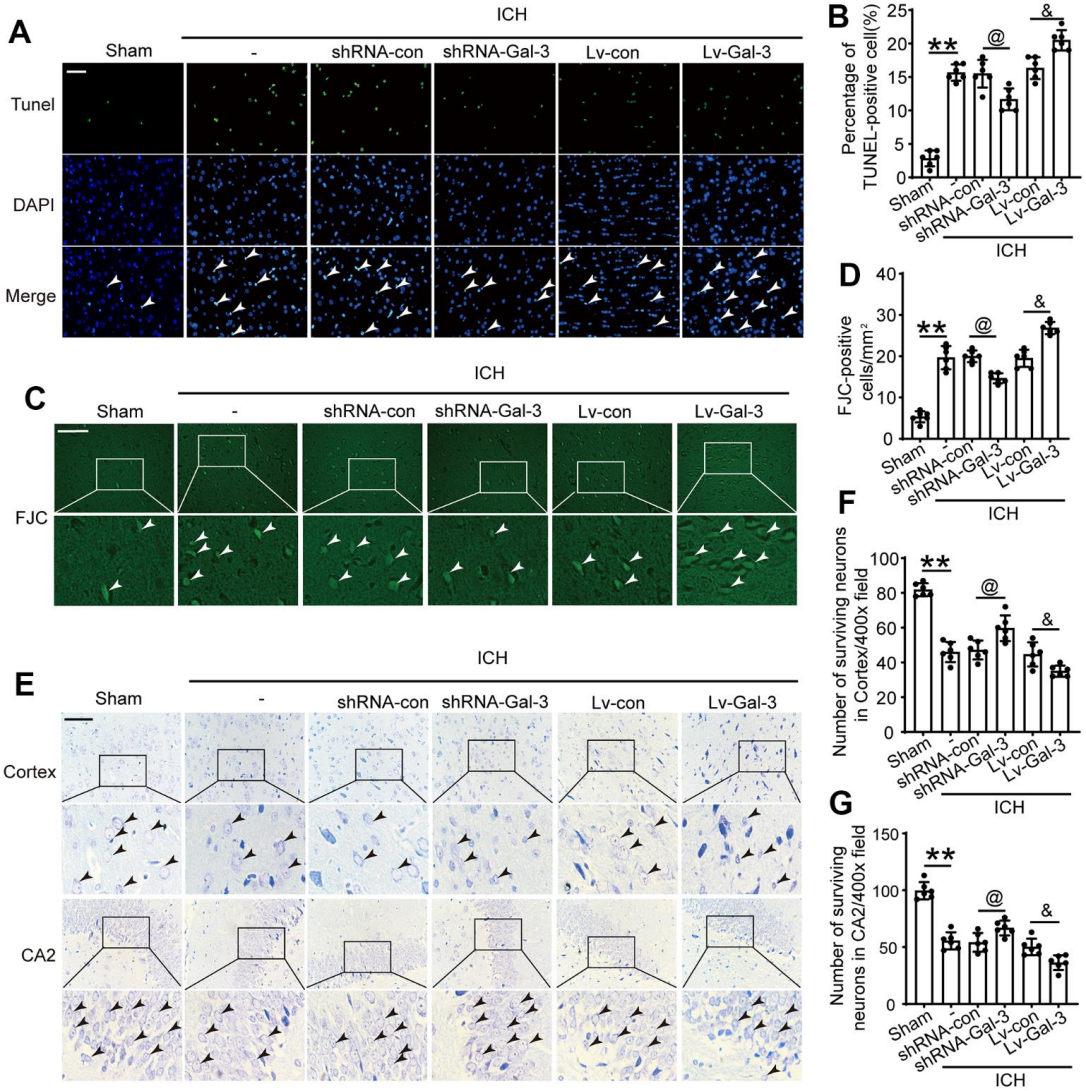
**Inhibition of Gal-3 reduced apoptosis and neuron loss induced by ICH.** (**A**, **B**) Apoptotic cells were labeled in brain sections using TUNEL staining, and the percentage of apoptotic cells was analyzed statistically. Arrow indicated TUNEL positive cells. Nuclei were labeled with DAPI (blue). (**C**, **D**) FJC staining. Arrow indicated FJC positive cells. (**E**–**G**) Nissl staining was used to assess the loss of neurons in the CA2 region of the hippocampus and in the cortex. Arrow indicated surviving cells. Scale bar = 50um. The black dots represent individual data in each group. **p < 0.01 and *p < 0.05 vs. Sham group, ^@^*p* < 0.05 vs. ICH+shRNA-con group, ^&^*p* < 0.05 vs. ICH+Lv-con group, n = 6.

Apoptosis, neuronal degeneration, and loss are crucial manifestations of brain damage caused by cerebral hemorrhage. Previous studies confirmed that Gal-3 plays a vital role in the post-ICH inflammatory response. Subsequently, we evaluated the involvement of Gal-3 in ICH-induced injury using TUNEL, FJB and Nissl staining. Initially, we observed cellular apoptosis in brain tissue surrounding the hematoma after ICH. Results indicated a significant increase in apoptosis following ICH compared to the sham group. This effect was more pronounced with Gal-3 overexpression, resulting in more severe apoptosis in the LV-Gal-3 group. In contrast, apoptosis was reduced by shRNA-Gal-3, with fewer apoptotic cells in the shRNA-Gal-3 group than the shRNA-Con group, suggesting Gal-3 also influences apoptosis after ICH (Figure 4A–4D). Subsequently, we conducted Nissl staining in the hippocampal CA2 area and cortex surrounding the hematoma. Experimental findings first demonstrated neuronal degeneration and loss following ICH, occurring in both the cortex and hippocampus. In the LV-Gal-3 group, we observed more severe neuronal loss, whereas in the shRNA-Gal-3 group, damage was mitigated and neuronal loss decreased (Figure 4E–4G). This implies that Gal-3 regulates neuronal degeneration and loss following ICH, and elevated Gal-3 levels after ICH can mediate more severe secondary brain damage.

### Downregulation of Gal-3 expression aids in alleviating LDH, blood-brain barrier (BBB) disruption, cerebral edema, and oxidative stress

**Figure 5:**
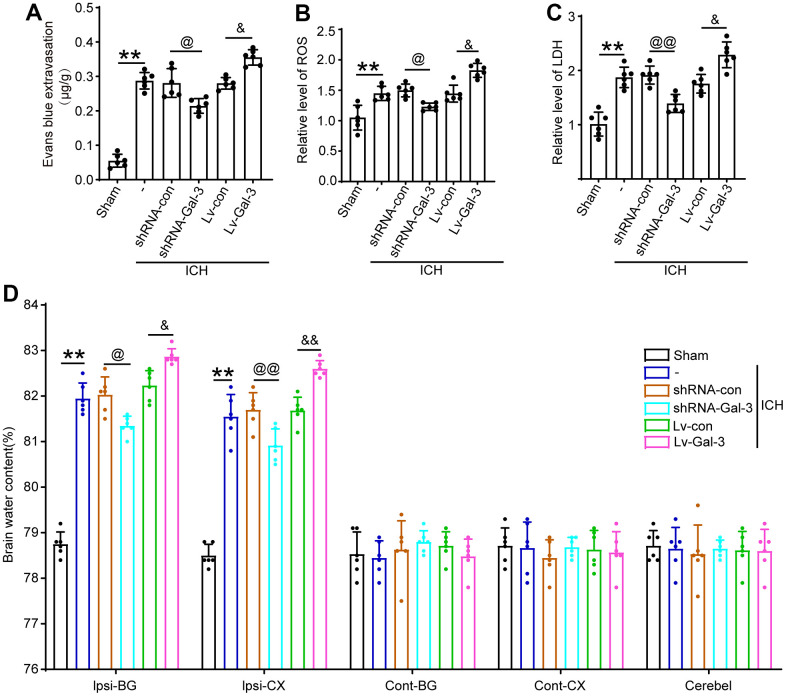
**Upregulation of Gal-3 increased the BBB impairment, LDH, oxidative stress reaction, brain edema induced by ICH.** (**A**) Evans blue extravasation. (**B**) ROS. (**C**) LDH. (**D**) Brain water content. The black dots represent individual data in each group. **p < 0.01 and *p < 0.05 vs. Sham group, ^@@^*p* < 0.01 and ^@^*p* < 0.05 vs. ICH+shRNA-con group, ^&&^*p* < 0.01 and ^&^*p* < 0.05 vs. ICH+Lv-con group, n = 6.

The BBB is a crucial indicator of SBI. Evans blue extravasation was employed to assess BBB permeability. The result showed that Gal-3 exacerbated BBB impairment in ICH rats (Figure 5A). By detecting ROS and LDH, we found that in the LV-Gal-3 group, the level of ROS and LDH were remarkable increased, whereas in the shRNA-Gal-3 group, the level of ROS and LDH were decreased (Figure 5B, 5C). To investigate the impact of Gal-3 on cerebral edema following ICH induction, brain water content was determined using the wet/dry weight method. Compared to the LV-Gal-3 group, brain water content was significantly worsened, while downregulating Gal-3 expression decreased cerebral edema (Figure 5D).

### Gal-3 promoted behavioral deficits induced by ICH

**Figure 6:**
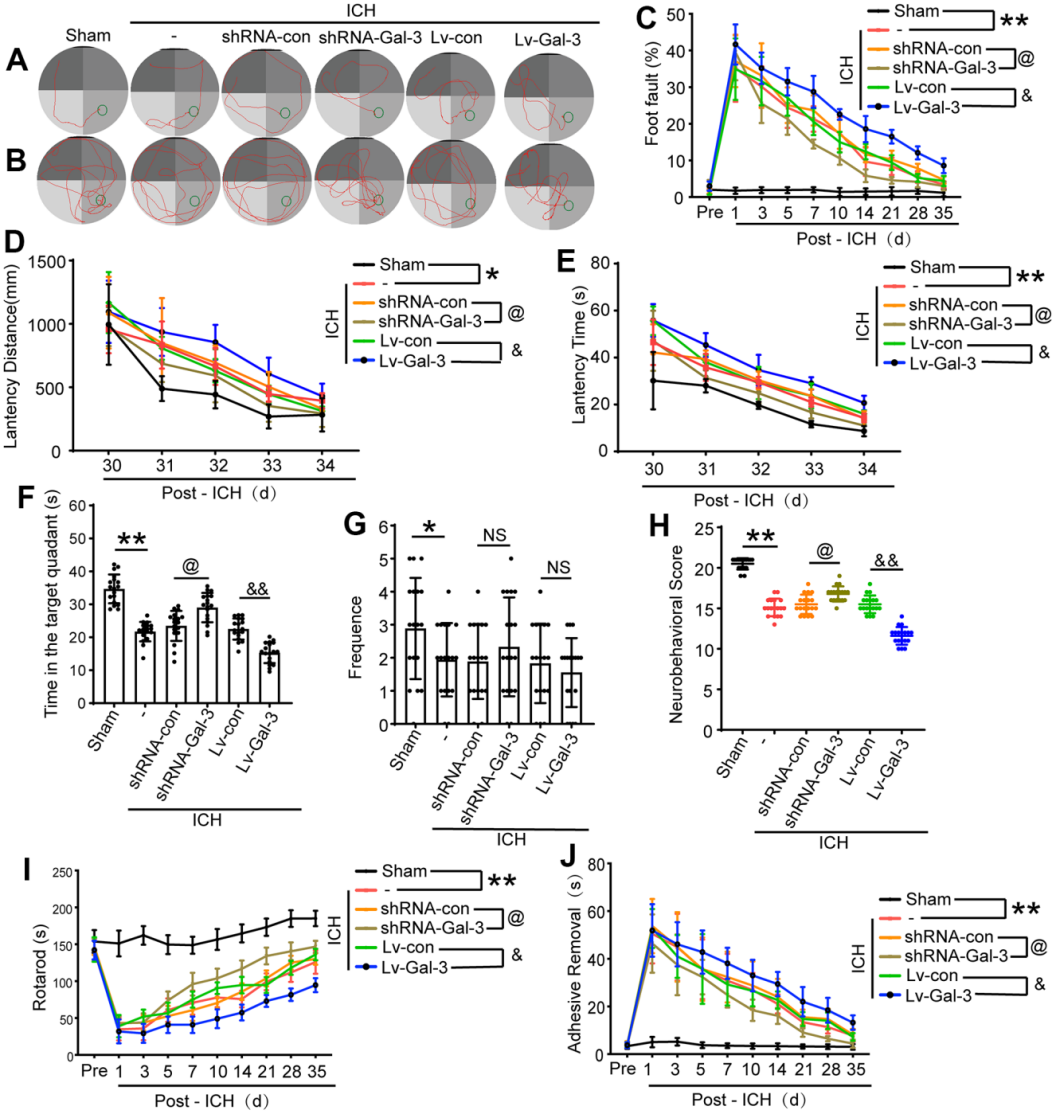
**Downregulation of Gal-3 can improve ethology deficits after ICH.** (**A**) Representative track of Morris water maze experiment during 29-34 days after ICH. (**B**) Representative track of Morris water maze experiment on 35 days after ICH. (**C**) Foot fault test. (**D**, **E**) Latency distance and time of Morris water maze experiment during 29-34 days after ICH. (**F**, **G**) Time in target quadrant and frequency cross the platform of Morris water maze experiment on 35 days after ICH. (**H**) Neurobehavioral score. Higher scores suggest less neurobehavioral deficits. (**I**) The motor coordination ability of rats after ICH was evaluated by rotarod test, long latency time indicates good motor coordination ability. (**J**) Adhesive-removal test. The black dots represent individual data in each group. **p < 0.01 and *p < 0.05 vs. Sham group, ^@^*p* < 0.05 vs. ICH+shRNA-con group, ^&&^*p* < 0.01 and ^&^*p* < 0.05 vs. ICH+Lv-con group, n = 18.

Ultimately, an array of behavioral assessments was employed to ascertain the influence of Gal-3 on the conduct of rats after ICH. On 3 days after ICH, we initially conducted a modified Garcia score (Figure 6H). The scores indicated that the rats exhibited pronounced behavioral deficits after ICH. With the overexpression of Gal-3, the scores were observed to be lower, implying exacerbated neurological damage. Conversely, in the shRNA-Gal-3 group, the scores displayed a slight improvement compared to the shRNA-Control group, suggesting partial resistance to the neural function impairment induced by ICH. Simultaneously, we executed a rotarod test to assess the motor capabilities of the rats. (Figure 6I). The results revealed that due to Gal-3 overexpression, the duration spent by the rats on the rod was significantly diminished, indicating poor motor coordination. However, following Gal-3 down-regulation, we observed a substantial prolongation of the rod-on time for the rats.

Furthermore, we performed foot fault and adhesive removal experiments to evaluate body balance and sensorimotor deficits. These assessments primarily observed the rate of missteps in the rat’s left forelimb and the time required to remove an adhesive. Initially, in comparison to the sham group, ICH injury led to a significant increase in the rat’s foot fault step rate and adhesive removal time, indicating damage to body balance and sensorimotor function. Subsequently, after Gal-3 overexpression, the rate of missteps and removal time were considerably increased compared to the negative virus group. However, in the shRNA-Gal-3 group that down-regulated Gal-3, the fault step rate and adhesive removal time were reduced, suggesting a mitigation of sensorimotor injury (Figure 6C, 6J). These experiments collectively demonstrated that Gal-3 plays a role in ICH-induced motor and sensory impairments.

Lastly, between 29 and 34 days after ICH, we conducted a Morris water maze experiment to evaluate the long-term spatial learning and memory capabilities of the rats. The results indicated that following ICH injury, the spatial learning and memory abilities of the rats were compromised. In comparison to the Sham group, though the swimming speeds of the two groups were analogous, the latency time for the rats increased, and the swimming distance to the platform became more extensive. In the shRNA-Gal-3 group, the damage was lessened, and the latency time and swimming distance were shortened. However, in cases of Gal-3 overexpression, the spatial learning and memory abilities were further impaired, and the latency time and swimming distance were significantly augmented (Figure 6A, 6D, 6E). The probe test conducted on day 35 after ICH also revealed that Gal-3 overexpression exacerbated spatial learning and memory impairment, and the proportion of time spent in the target quadrant by the rats was considerably reduced. In contrast, in the sh-RNA-Gal-3 group that down-regulated Gal-3, the proportion of time spent in the target quadrant increased (Figure 6B, 6F, 6G). These experimental findings suggest that Gal-3 also participates in long-term spatial learning and memory impairment after ICH.

## DISCUSSION

Intracerebral hemorrhage (ICH) poses a significant risk to people’s health worldwide [30]. ICH results in a complex mechanism of central nervous system injury, including both physical injury and primary injury as a result of hematoma formation. Secondary injury primarily arises from microglial activation, mitochondrial dysfunction, and the release of toxic neurotransmitters and inflammatory mediators [29]. As crucial innate immune cells, microglia serve as the brain’s sentinels and are believed to be the first non-neuronal cells to respond to acute brain injury [31–33]. Microglia not only contribute to hematoma and edema absorption after ICH but also play a vital role in mitigating inflammatory brain injury and expediting neurological function recovery after ICH [34]. Brain injury triggers microglial activation to a pro-inflammatory phenotype (M1), leading to the infiltration of various immune cells from the bloodstream, subsequently causing the release of inflammatory factors, chemokines, free radicals, and other toxic substances. These molecules result in exacerbated lymphocyte infiltration and a sustained inflammatory response cycle, accelerating cell death in the central nervous system and exacerbating brain injury [35]. Roughly a week after ICH, certain factors drive microglia to transition from a pro-inflammatory to an anti-inflammatory phenotype (M2), secreting various protective factors to inhibit the inflammatory response and promote self-repair of injured brain tissue [36]. Thus, modulating microglial phenotypic transformation and inhibiting neuroinflammatory response may be a crucial strategy for treating secondary brain injury after ICH [37].

Gal-3, a unique cell-connecting molecule and cellular bridge, can bind to a wide array of substrates, including signaling molecules, transcriptional regulators, ribonucleoproteins, cell surface receptors, and matrix proteins [38, 39], which influences numerous processes such as proliferation, migration, apoptosis, fibrosis, and inflammation [40–44]. Concurrently, Gal-3 can be highly expressed in various cell types, including macrophages [45, 46], fibroblasts [47] and cancer cells [48]. We therefore suggest that Gal-3 has the potential to regulate a variety of cellular processes.

In the initial experimental phase, Western blot results demonstrated a gradual increase in Gal-3 protein expression following ICH, peaking approximately 24 hours after ICH before gradually decreasing. This implies that Gal-3 may play a role in ICH, although its specific function remains unknown. Immunofluorescence staining revealed that Gal-3 was primarily expressed in microglia. Since microglia are the central nervous system’s primary immune cells and the most critical biological function of Gal-3 is participating in immune responses, it can be inferred that Gal-3 modulates the inflammatory response by influencing microglial function through specific signaling pathways after ICH; however, the specific role of Gal-3 necessitates further experimentation.

First, we effectively regulated the expression of Gal-3 by means of interference. Western blot and immunofluorescence assays revealed the intervention agent effectively governed Gal-3 protein expression levels. After ICH, a Co-IP experiment revealed that Gal-3 played a related role by interacting with TLR-4, and this complex was responsible for the subsequent chain of physiological and pathological processes. IF co-staining showed that elevated Gal-3 levels correlated with increased M1 microglia populations. We deduced that Gal-3 stimulates inflammatory responses by facilitating microglial conversion to the M1 phenotype during the acute phase of ICH. Given the altered microglial phenotype, associated inflammatory factor levels must also adjust. We selected TNF-α, IL-1β and IL-6 as representative cytokines for measurement standards and determined each group’s cerebrospinal fluid and serum inflammatory factor content via ELISA. Results indicated increased Gal-3 expression correlated with elevated pro-inflammatory factors, such as TNF-α, IL-1β, and IL-6, in CSF and serum. Because the phenotype transformation of M1 type microglia and the changes in the levels of related inflammatory factors occur at the same time when the expression level of Gal-3 changes, this phenomenon of simultaneous changes can further explain that the number of M1 type microglia increases when the expression level of Gal-3 increases. Combined with the results of IP, Gal-3 can induce Microglia to transform into M1 phenotype by combining with TLR-4, thus controlling inflammatory reaction. Through FJB staining, TUNEL staining, and Nissl staining, we can observe that when the expression level of Gal-3 increases, there are more dead cells in the brain tissue than in the control group. However, when the expression level of Gal-3 decreases, the number of dead cells decreases accordingly. This result indicates that inhibiting the expression of Gal-3 can effectively reduce brain cell death after ICH. In connection with the impact of Gal-3 on inflammatory response, we speculate that on the one hand, Gal-3/TLR-4 complex increases inflammatory cell death caused by inflammation by amplifying the inflammatory response, and on the other hand, Gal-3/TLR-4 complex may also promote cell death through other non-inflammatory mechanisms, and the non-inflammatory effects of Gal-3 also needs worth further exploration in our future work. Subsequent brain injury experiments demonstrated Gal-3 overexpression expedited blood-brain barrier disruption, cerebral edema, and oxidative stress. The above molecular level experiments have demonstrated that Gal-3/TLR-4 can promote inflammatory response and amplify brain damage after ICH. However, elucidating the function of Gal-3 at the molecular level still lacks persuasiveness, so it is necessary to conduct short-term and long-term behavioral verification at the individual level. Short-term neurological scores revealed ICH rats with suppressed Gal-3 expression outperformed the control group. Reduced Gal-3 expression levels correlated with faster limb function recovery in ICH rats, suggesting Gal-3’s detrimental role after ICH is reflected behaviorally. Long term behavioral experiments further validate the effect of Gal-3 on long-term outcomes in rats with ICH. Prolonged rotarod testing confirmed post-ICH motor ability recovery, with the shRNA-Gal-3 group spending more time on the apparatus than control group. This result signified diminished Gal-3 protein expression effectively alleviated ICH-induced brain injury. Additionally, adhesive removal and foot fault tests demonstrated that decreasing Gal-3 expression levels was conducive to sensory and balance function recovery. Lastly, Morris water maze tests conducted 29-34 days after ICH assessed platform discovery time and swim trajectory, exploring learning and memory ability recovery rates among rat groups. The shRNA-Gal-3group exhibited a substantially reduced platform discovery time and swim distance, implying that decreasing Gal-3 expression promotes learning ability recovery in this rat population, ultimately mitigating ICH-induced brain damage. Behavioral experiments have shown that Gal-3 inhibits the recovery of neural function in rats with ICH, so inhibiting Gal-3 expression can effectively promote the recovery of defective function in experimental animals, which is consistent with the results of our previous molecular level experiments. Therefore, the results of this series of behavioral experiments all show that reducing the expression level of Gal-3/TLR-4 contributes to the recovery of rat behavior, which is consistent with the results of molecular experiments. The overall conclusion shows that reducing the expression of Gal-3 can help to reduce CNS inflammatory reaction, reduce cell death, reduce brain edema, protect the integrity of the Blood–brain barrier, and alleviate the damage of ICH to the central nervous system, reflected at the individual level, it can promote the recovery of neural function.

In the future, we will focus on the mechanism of action of Gal-3, and relevant reports will focus on the Gal-3/TLR-4 signaling pathway. We anticipate that the functional pathways of Gal-3 will soon be fully understood and integrated into clinical applications.

## CONCLUSIONS

Through this sequence of investigations, we ascertain that diminishing Gal-3/TLR-4 complex expression can suppress inflammation, reduce cellular mortality, and mitigate cerebral tissue damage resulting from secondary brain injury after ICH. Concurrently, Gal-3/TLR-4 complex can decelerate recovery in motor, sensory, learning, and memory capabilities in rats following ICH impairment, which also corresponds to the biological impact of Gal-3 in promoting inflammatory response, amplifying neuronal cell death, and delaying the restoration of injured brain tissue at an individual level. Inhibition of Gal-3 expression proves beneficial for ICH treatment. This molecular pathway may provide a novel perspective for clinical intervention, aiming to enhance the long-term prognosis of patients suffering from ICH.
